# Automatic Adjustment of the Inspiratory Trigger and Cycling-Off Criteria Improved Patient-Ventilator Asynchrony During Pressure Support Ventilation

**DOI:** 10.3389/fmed.2021.752508

**Published:** 2021-11-12

**Authors:** Ling Liu, Yue Yu, Xiaoting Xu, Qin Sun, Haibo Qiu, Davide Chiumello, Yi Yang

**Affiliations:** ^1^Jiangsu Provincial Key Laboratory of Critical Care Medicine, Department of Critical Care Medicine, Zhongda Hospital, School of Medicine, Southeast University, Nanjing, China; ^2^SC Anesthesia and Resuscitation, San Paolo Hospital—University Campus, ASST Santi Paolo e Carlo, Milan, Italy; ^3^Department of Health Sciences, University of Milan, Milan, Italy; ^4^Coordinated Research Center of Respiratory Insufficiency, University of Milan, Milan, Italy

**Keywords:** automatic adjustment system, pressure support ventilation, patient-ventilator asynchrony, cycling-off, trigger

## Abstract

**Background:** Patient-ventilator asynchrony is common during pressure support ventilation (PSV) because of the constant cycling-off criteria and variation of respiratory system mechanical properties in individual patients. Automatic adjustment of inspiratory triggers and cycling-off criteria based on waveforms might be a useful tool to improve patient-ventilator asynchrony during PSV.

**Method:** Twenty-four patients were enrolled and were ventilated using PSV with different cycling-off criteria of 10% (PS_10_), 30% (PS_30_), 50% (PS_50_), and automatic adjustment PSV (PS_AUTO_). Patient-ventilator interactions were measured.

**Results:** The total asynchrony index (AI) and NeuroSync index were consistently lower in PS_AUTO_ when compared with PS_10_, PS_30_, and PS_50_, (*P* < 0.05). The benefit of PS_AUTO_ in reducing the total AI was mainly because of the reduction of the micro-AI but not the macro-AI. PS_AUTO_ significantly improved the relative cycling-off error when compared with prefixed controlled PSV (*P* < 0.05). PS_AUTO_ significantly reduced the trigger error and inspiratory effort for the trigger when compared with a prefixed trigger. However, total inspiratory effort, breathing patterns, and respiratory drive were not different among modes.

**Conclusions:** When compared with fixed cycling-off criteria, an automatic adjustment system improved patient-ventilator asynchrony without changes in breathing patterns during PSV. The automatic adjustment system could be a useful tool to titrate more personalized mechanical ventilation.

## Introduction

Pressure support ventilation (PSV) is the most widely used partial mode of assistance to minimize the effort of patients in breathing. During PSV, the assist is delivered by means of a pneumatic signal generated by patient effort and measured in the ventilatory circuit, i.e., flow or pressure (1). The ventilator usually cycles from inspiration to expiration when the inspiratory flow falls to a predetermined fraction of the peak inspiratory flow, which is the cycling-off criterion (2). Ideally, the ventilator trigger and cycling should coincide with the beginning and the end of the inspiratory effort of the patients (3). However, patient-ventilator asynchrony is common during PSV (4, 5), thereby contributing to the increased patient effort, increased duration of mechanical ventilation, and even increased mortality (6).

During PSV, prefixed pneumatic controllers can become progressively less effective, especially when patients have abnormal respiratory mechanics or ventilator over-assist (7). Delayed or missed triggers are sensed as an uncomfortable isometric load leading to increased effort intensity and pronounced dyspnea (8). Moreover, with prefixed cycling-off criteria, such as the default value of 30% peak flow in some ventilators, premature cycling is more frequent in patients with restrictive breathing patterns characterized by low respiratory system compliance and may result in double triggering. Delayed cycling occurs more frequently in patients with an obstructive pattern characterized by high resistance (6, 9). Different approaches for optimal ventilator triggering and cycling have been developed to minimize these problems, such as flow-triggering sensitivity and adjustable flow cycling during PSV.

It has been demonstrated that a noninvasive method based on flow and airway-pressure tracings was effective for detecting asynchrony (10–12). Therefore, an automatic adjustment system (IntelliCycleTM2.0) capable of automatically adjusting, breath by breath, the triggering and cycling-off criteria based on pressure-time and flow-time waveforms during PSV have been developed (see Supplementary Material).

The objective of our study was to show a reduction in patient-ventilator asynchrony with the use of an automatic adjustment system as compared with prefixed trigger and cycling-off criteria in patients with PSV.

## Methods

This unblinded crossover study was conducted in a 60-bed general intensive care unit of a teaching hospital affiliated with Southeast University in China. The protocol was approved by the Institutional Ethics Committee of Zhongda Hospital (number 2016ZDSYLL067-P01). Written informed consent was obtained from the legal primary decision-maker, which was the spouse of the patient or the parent or child if no spouse. The trial was registered at clinicaltrials.gov (NCT04091269).

### Patients

Postoperative (abdominal surgery or orthopedic surgery) or acute respiratory failure patients were eligible when meeting all the following criteria: receiving invasive mechanical ventilation and being able to sustain PSV more than 1 h with inspiratory support ≤ 15 cm H_2_O. Patients were excluded if (1) age <18 or >85 years; (2) tracheostomy at time of the study; (3) sedation level on the Richmond Agitation–Sedation Scale ≤ −2 or ≥ 2; (4) contraindication for nasogastric tube insertion, e.g., history of esophageal varices, gastroesophageal surgery in the previous 12 months, or gastroesophageal bleeding in the previous 7 days, international standard ratio > 1.5, activated partial thromboplastin time > 44 s, history of leukemia (13); and (5) hemodynamic instability (heart rate > 140 beats/min, vasopressors required with ≥ 5 μg/kg/min dopamine/dobutamine, or ≥ 0.2 μg/kg/min norepinephrine).

### Study Protocol

After obtaining consent, enrolled patients were switched to a Servo-i ventilator (Maquet, Solna, Stockholm, Sweden). A 16-F nasogastric feeding tube (NeuroVent Research Inc., Toronto, ON, Canada) with electrodes measuring the electrical activity of the diaphragm (EAdi) and balloons measuring esophageal (Pes) pressures was inserted through the nose and secured after confirming positioning according to the recommendations of the manufacturer. Static respiratory system compliance (*C*_*RS*_), resistance (*R*_*RS*_), and intrinsic positive end-expiratory pressure (PEEPi) were measured during volume control ventilation (without spontaneous drive) (see Supplementary Material).

Then sedation was decreased to maintain light sedation with the Richmond Agitation–Sedation Scale ranging from 0 to −2. As spontaneous breathing and EAdi recovered, patients were switched to an SV800 ventilator with IntelliCycleTM2.0 which can automatically adjust triggering and cycling-off criteria breath-by-breath, (Mindray, Shenzhen, China) and were ventilated by PSV with the pressure support level adjusted to a target tidal volume (V_T_) of 6 ml/kg (of predict body weight, PBW). During the entire recording period, PEEPe and a fraction of inspired oxygen (FiO_2_) were maintained as set by the clinician in charge of the patient.

During prefixed pneumatically controlled PSV, the inspiratory trigger was set at 1.5 L/min for flow triggering, and the rate of rise in pressure was set to 0.05 s in all patients. The cycling-off criteria were set to 10% (PS_10_), 30% (PS_30_), and 50% (PS_50_). During automatic adjustment PSV, the inspiratory trigger was set as flow-trigger 1.5 L/min, the rate of rise in pressure was set to 0.05 s, and the cycling-off criterion was set to “AUTO” (PS_AUTO_). Both the trigger and cycling-off criteria were adjusted by the automatic adjustment system according to an established algorithm based on the pressure-time and flow-time waveforms (Supplementary Figures 1, 2). First, patients were ventilated with four independent modes (PS_10_, PS_30_, PS_50_, and PS_AUTO_) applied in randomized order (Supplementary Table 1). Each independent condition was maintained for 20 min without washout periods (Supplementary Figure 3).

### Data Acquisition and Analysis

Flow, airway pressure (P_aw_), esophageal pressure (P_es_), and EAdi were acquired during the 20-min time window in each condition at 100 Hz from the ventilator *via* an RS 232 interface connected to a computer. Data were stored for later offline analysis (NeuroVent Research Inc., Toronto, ON, Canada). To quantify patient-ventilator interaction, all variables were calculated manually breath by breath from a stable 3-min period in each condition using customized software (NeuroVent Research Inc., Toronto, ON, Canada) by two independent researchers who were blinded to the patient number and assigned order of crossover treatments, and mean values were calculated. In the event of a mismatch, a third researcher was consulted.

Six types of asynchrony were analyzed as previously described by Thille et al. and Lamouret et al. (6, 14). Macro asynchronies include ineffective triggering, which is defined by the existence of a diaphragmatic signal without a respiratory cycle; auto-triggering is defined by the existence of a ventilator cycle without a diaphragmatic signal; and double triggering is defined by the presence of two successive inspiratory cycles without an intermediate expiration or with an interrupted expiration. Micro-asynchronies are defined by a time difference exceeding 200 ms between the onset of the EAdi and the early initial rise in Paw; between the 70% of peak EAdi and early decrease in airway pressure (the opening of the expiratory valve)-late cycling; and between the decrease in airway pressure and 70% of peak EAdi-premature cycling. For each subtype of asynchrony, a percentage of asynchronies was calculated as follows: the number of asynchrony events divided by the total neural respiratory rate (which corresponds to the total EAdi signals) × 100%. Macro-asynchrony index (AI), micro-AI, and total AI were calculated as the number of macro asynchrony events, micro-asynchrony, or total asynchrony events divided by the neural respiratory rate × 100%.

Triggering and cycling-off errors, which were classified as either too late (positive values) or too early (negative values) (13), breathing pattern, inspiratory effort, and inspiratory effort for triggering were measured (see Supplementary Material). To estimate the overall extent of asynchrony and dys-synchrony, the NeuroSync index was calculated by averaging the percentage errors in triggering and cycling-off for all breaths (13). The primary endpoint was the difference in the total AI between PS_AUTO_ and PSV with prefixed triggering and cycling-off criteria (PS_10_, PS_30_, and PS_50_).

### Statistical Analysis

All statistical analyses were carried out using SPSS 20 (Chicago, IL, USA). The values are stated as mean ± SD unless specified otherwise. Data from two *post-hoc* subgroups, a restrictive subgroup defined as having C_RS_ < 40 ml/cm H_2_O with *R*_*RS*_ < 12 cm H_2_O/LS, and an obstructive subgroup, defined as having *R*_*RS*_ > 12 cm H_2_O/LS with C_RS_ > 40 ml/cm H_2_O, were analyzed. The normal distribution of continuous variables was assessed by using the Shapiro–Wilk test. Log-transformation was used for skewed data. Variables were compared between modes using repeated-measures ANOVA followed by Bonferroni's *post-hoc* test. Categorical data were compared by the chi-square test followed by Bonferroni's *post-hoc* test. *P*-values <0.05 were considered significant.

## Results

The study included 24 patients, such as eight patients in the restrictive subgroup, eight patients in the obstructive subgroup, and eight other patients without obvious acute respiratory failure (C_RS_ > 40 ml/cm H_2_O with *R*_*RS*_ < 12 cm H_2_O/LS). Patient characteristics and lung mechanism are summarized in Table 1.

**Table 1:** Patient characteristics.

**Parameter**	**All (*n* = 24)**	**Obstructive subgroup (*n* = 8)**	**Restrictive subgroup (*n* = 8)**	**Other patients (*n* = 8)**
Sex, male/female	19/5	5/3	7/1	7/1
Age, year	68 ± 17	75 ± 9	65 ± 17	68 ± 23
APACHE II	17.1 ± 5.3	17.9 ± 4.0	18.7 ± 6.0	14.8 ± 6.5
Main diagnosis				
Pneumonia, *n* (%)	4 (16.7%)	–	4 (50%)	
Extrapulmonary sepsis, *n* (%)	2 (8.3%)	–	2 (25%)	
AECOPD, *n* (%)	8 (33.3%)	8 (100.0%)	–	
Abdominal surgery	4 (16.7%)	–	–	4 (50.0%)
Orthopedic surgery, *n* (%)	4 (16.7%)	–	–	4 (50.0%)
Severe trauma, *n* (%)	2 (8.3%)		2 (25%)	
RASS	0 (−1, 0)	0 (−1, 0)	−1 (−2, 0)	0 (−1, 0)
PBW, Kg	63 ± 8	63 ± 7	59 ± 9	65 ± 7
PaO_2_, mm Hg	107 ± 36	96 ± 30	95 ± 16	135 ± 42
PaO_2/_FiO_2_	276 ± 90	362 ± 78	239 ± 76	227 ± 41
PaCO_2_, mm Hg	39 ± 11	48 ± 14	36 ± 6	32 ± 4
pH	7.41 ± 0.06	7.39 ± 0.05	7.43 ± 0.03	7.41 ± 0.06
C_RS_, ml/cm H_2_O	45.8 ± 9.7	50.9 ± 5.8	34.1 ± 5.1	52.5 ± 3.5
R_RS_, cm H_2_O/L/S	12.1 ± 4.9	17.9 ± 4.1	9.2 ± 2.1	9.4 ± 1.7
PEEPi, cm H_2_O	1.7 ± 2.0	3.6 ± 2.4	0.9 ± 0.2	0.7 ± 0.7

*Data are provided as mean ± SD or median (interquartile range)*.

*APACHE II, Acute Physiology and Chronic Health Evaluation II; RASS, Richmond Agitation-Sedation Scale; PBW, predictive body weight; C_RS_, static compliance of the respiratory system; R_RS_, resistance of respiratory system; PEEPi, static intrinsic positive end expiratory pressure*.

### AI

**Figure 1:**
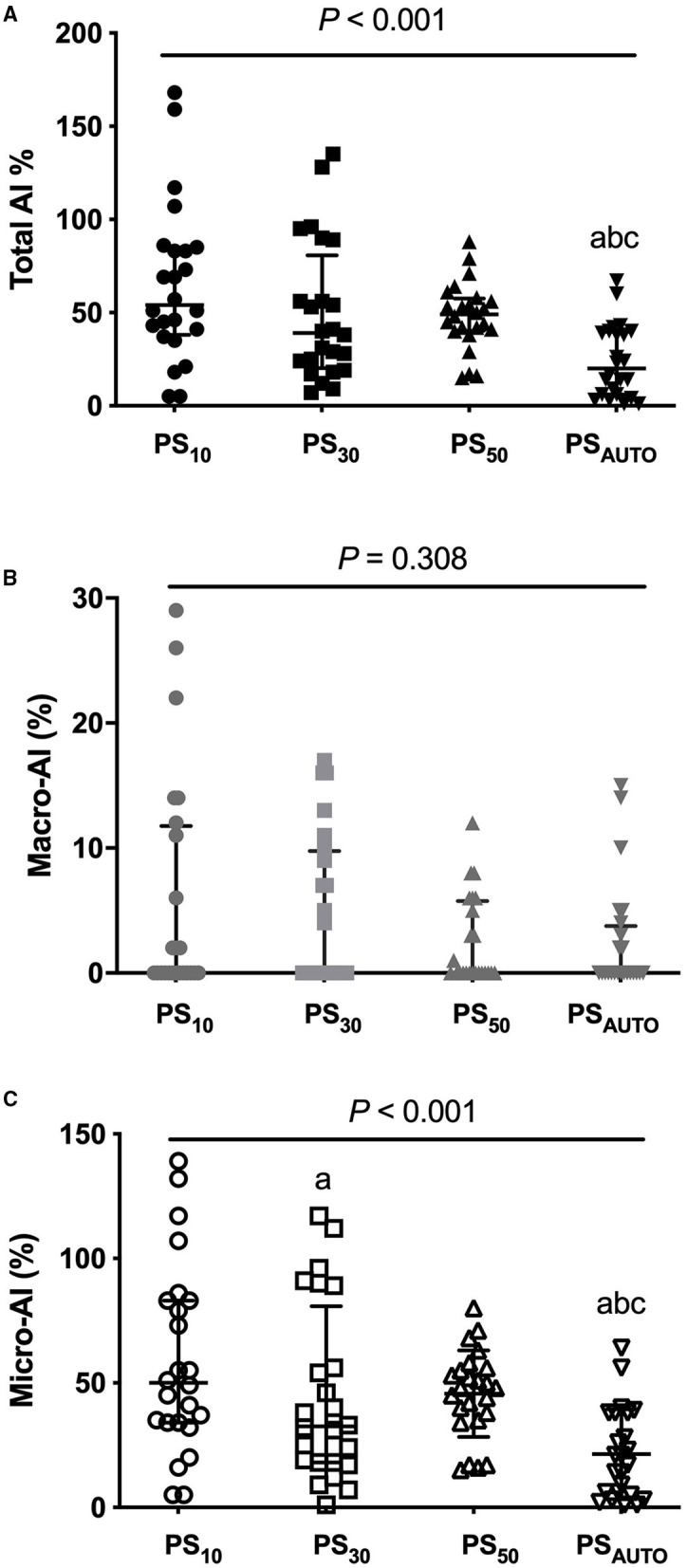
Total AI **(A)**, macro-AI **(B)**, and micro-AI **(C)** in different modes. AI, asynchrony index; PS_10_, pressure support ventilation with cycling-off criteria set to 10%; PS_30_, pressure support ventilation with cycling-off criteria set to 30%; PS_50_, pressure support ventilation with cycling-off criteria set to 50%; PS_AUTO_, pressure support ventilation with automatic. Gray lines showed median (interquartile range). Compared with PS_10_, ^a^*P* < 0.05; Compared with PS_30_, ^b^*P* < 0.05; compared with PS_50_, ^c^*P* < 0.05.

Total AI was consistently lower in PS_AUTO_ when compared with PS_10_, PS_30_, and PS_50_, (*P* < 0.05). The benefit of PS_AUTO_ in reducing total AI was mainly in the reduction of micro-AI but not macro-AI (Figure 1). The percentages of all kinds of asynchronies are reported in Table 2. Total AI and micro-AI were lower in PS_AUTO_ when compared with PS_10_ and PS_30_ in the obstructive subgroup and were lower in PS_AUTO_ when compared with PS_50_ in the restrictive subgroup (Supplementary Table 2).

**Table 2:** Asynchronies, NeuroSync index, inspiratory effort, and relative timing errors of cycling-off and trigger in different modes.

**Parameters**	**PS_**10**_**	**PS_**30**_**	**PS_**50**_**	**PS_AUTO_**	***P*** **value**
Ineffective triggering, %	0.0 (0.0, 2.3)	0.0 (0.0, 0.0)	0.0 (0.0, 1.0)	0.0 (0.0, 1.4)	0.118
Auto-triggering, %	0.0 (0.0, 0.0)	0.0 (0.0, 3.3)	0.0 (0.0, 0.0)	0.0 (0.0, 0.0)	0.039
Double triggering, %	0.0 (0.0, 6.7)	0.0 (0.0, 0.0)	0.0 (0.0, 0.0)	0.0 (0.0, 0.0)	0.569
Premature cycling-off, %	0.0 (0.0, 2.6)	0.0 (0.0, 1.8)	5.4 (0.0, 22.5)^b^	0.0 (0.0, 1.8)^c^	<0.001
Late cycling-off, %	7.1 (0.0, 28.1)	1.8 (0.0, 20.8)	0.0 (0.0, 0.0)^a^	0.0 (0.0, 0.0)^a^	<0.001
Inspiratory trigger delay, %	38.3 (22.4, 48.9)	25.9 (11.4, 47.1)	30.3 (16.3, 45.6)	19.0 (5.0, 31.3)^abc^	<0.001
NeuroSync index, %	15.3 ± 8.2	13.3 ± 6.7^a^	13.1 ± 4.8	9.7 ± 4.4^abc^	<0.001
“Perfect” synchrony breath, %	18.5 (16.4, 20.7)	21.9 (19.5, 24.2)^a^	19.7 (17.5, 21.9)	42.2 (39.5, 44.9)^abc^	<0.001
“Acceptable” synchrony breath, % (95% CI)	81.1 (78.9, 83.2)	87.9 (86.0, 89.7)^a^	89.5 (87.8, 91.2)^a^	94.8 (93.5, 96.0)^abc^	<0.001
PTP_es−Trig_, cmH_2_O.S.min^−1^	−3.1 (−6.0, −1.1)	−2.3 (−4.1, −1.1)	−2.0 (−3.6, −1.1)^a^	−1.9 (−3.7, −0.8)^ab^	<0.001
PTP_es_, cmH_2_O.S.min^−1^	−17.1 (−88.2, −13.1)	−38.7 (−71.3, −10.6)	−40.8 (−58.0, −8.9)	−37.4 (−61.2, −9.1)	0.802

*Data are provided as mean ± SD or median (interquartile range)*.

*NeuroSync index is an overall indicator of patient-ventilator interaction, where 0% error, perfect; 100% error, zero patient-ventilator interaction; PTP_es−Trig_, Pre-trigger Pes-time product; PTP_es_, Pes-time product; PS_10_, pressure support ventilation with cycling-off criteria set to 10%; PS_30_, pressure support ventilation with cycling-off criteria set to 30%; PS_50_, pressure support ventilation with cycling-off criteria set to 50%; PS_AUTO_, pressure support ventilation with automatic adjustment system; CI, Confidence interval, “perfect” synchrony, relative timing errors of triggering and for cycling-off ≤10% of neural timings, “acceptable” synchrony, relative timing errors of triggering and for cycling-off ≤10% of neural timings*.

*Compared with PS_10_*,

a *P < 0.05; Compared with PS_30_*,

b *P < 0.05; Compared with PS_50_*,

c *P < 0.05*.

### NeuroSync Index

**Figure 2:**
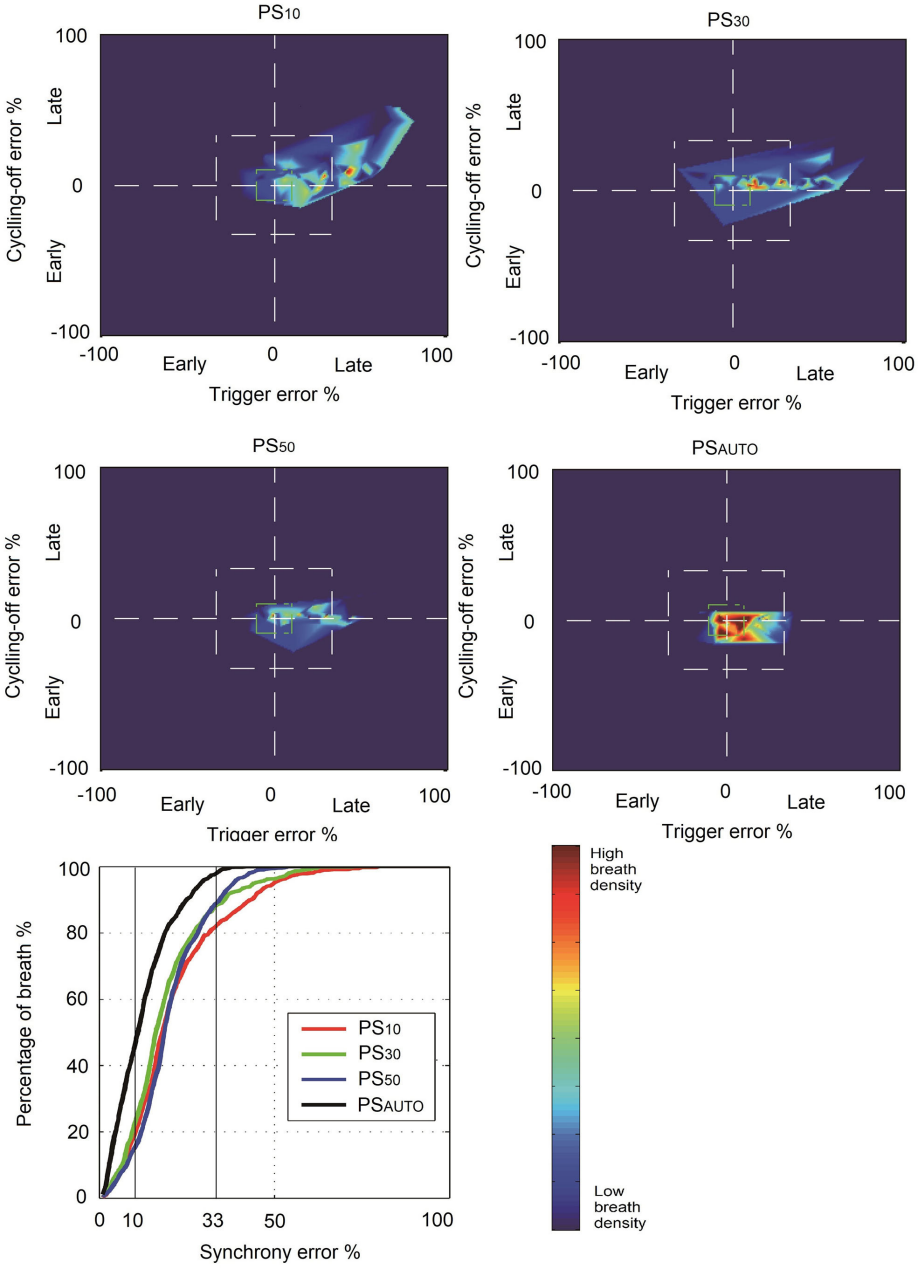
Breath density graph for relative trigger (*X*-axis) and cycling-off (*Y*-axis) errors, for all breaths in all patients, during each ventilator mode. PS_10_, pressure support ventilation with cycling-off criteria set to 10%; PS_30_, pressure support ventilation with cycling-off criteria set to 30%; PS_50_, pressure support ventilation with cycling-off criteria set to 50%; PS_AUTO_, pressure support ventilation with automatic adjustment system; asynchrony error, breathes inside the box of percentage error of neural timings.

The NeuroSync index (average of the percentage errors of triggering and cycling-off) was consistently lower in PS_AUTO_ when compared with PS_10_, PS_30_, and PS_50_, indicating improved patient-ventilator interaction (Table 2). Figure 2 shows a plot of the percentage errors of triggering (*X*-axis) and cycling-off (*Y*-axis) for every breath. We have inserted a small centered box suggesting “perfect” asynchrony to be ≤10% of neural timing and a larger box suggesting “acceptable” asynchrony to be ≤33% of neural timing (15). There were more “Perfect” asynchrony breaths and “Acceptable” asynchrony breaths in PS_AUTO_ than in the fixed cycling-off criteria mode (PS_10_, PS_30_, and PS_50_, all *P* < 0.05; Figure 2).

### Cycling-Off and Triggering Error

**Figure 3:**
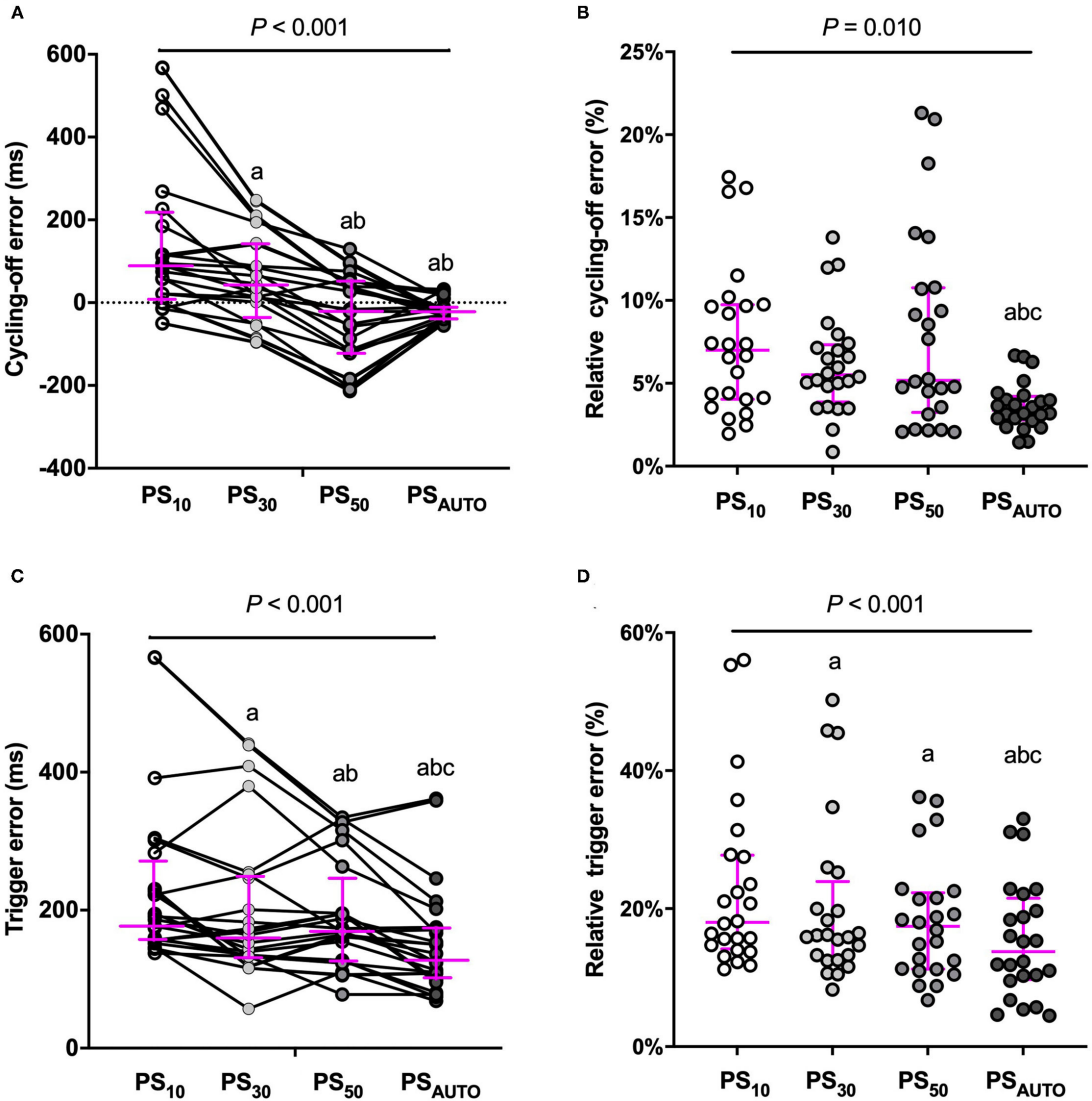
Cycling-off error and trigger error in different modes. **(A)** cycling-off error, **(B)** relative cycling-off error, **(C)** trigger error, and **(D)** relative trigger error, *Y*-axis for cycling-off error: positive values indicate late cycling-off, and negative values indicate early cycling off. Magenta line showed median (interquartile range). ms, millisecond; PS_10_, pressure support ventilation with cycling-off criteria set to 10%; PS_30_, pressure support ventilation with cycling-off criteria set to 30%; PS_50_, pressure support ventilation with cycling-off criteria set to 50%; PS_AUTO_, pressure support ventilation with automatic. Compared with PS_10_, ^a^*P* < 0.05; Compared with PS_30_, ^b^*P* < 0.05; Compared with PS_50_, ^c^*P* < 0.05.

Automatic adjustment PSV significantly improved the relative cycling-off error when compared with PS_10_, PS_30_, and PS_50_ in the whole population (Figure 3). The relative cycling-off error in PS_AUTO_ was comparable with that in PS_50_ in the obstructive subgroup and was comparable with that in PS_10_ in the restrictive subgroup. PS_AUTO_ significantly shortened the absolute and relative triggering errors when compared with a prefixed trigger (PS_10_, PS_30_, or PS_50_; Figure 3). The Absolute and relative triggering errors were significantly lower when compared with PS_10_, PS_30_, and PS_50_ in the obstructive subgroup but not in the restrictive subgroup (Supplementary Figure 4).

### Respiratory Drive and Breathing Pattern

Inspiratory effort for triggering determined by PTP_es−trig_ was significantly lower in PS_AUTO_ when compared in PS_10_ and PS_30_; however, total inspiratory effort determined by PTP_es_ was not different among modes (Table 3). In the obstructive subgroup, PTP_es−trig_ was significantly lower in PS_AUTO_ than in PS_10_, PS_30_, and PS_50_ (*P* < 0.05; Supplementary Table 2). Peak airway pressure was higher in PS_10_ than in other modes. There was no difference in the respiratory drive between modes (Table 3). Breathing patterns and respiratory drive in obstructive and restrictive subgroups are shown in Supplementary Table 3.

**Table 3:** Breathing pattern and respiratory drive in different modes.

**Parameter**	**PS_**10**_**	**PS_**30**_**	**PS_**50**_**	**PS_**AUTO**_**	***P*** **value**
Ppeak, cmH_2_O	16.5 ± 4.2	15.4 ± 4.2^a^	15.4 ± 4.1^a^	14.6 ± 4.1^a^	<0.001
PEEP, cmH_2_O	6.2 ± 1.7	6.1 ± 1.6	6.3 ± 1.5	6.6 ± 1.5	0.120
Vt, cmH_2_O/kg	6.1 ± 0.2	6.1 ± 0.2	6.1 ± 0.3	6.0 ± 0.1	0.169
RR_N_, breath/min	20.1 ± 5.6	19.3 ± 7.7	19.3 ± 5.9	20.5 ± 6.4	0.331
Ti_N_, s	1.2 ± 0.0	1.1 ± 0.0	1.2 ± 0.0	1.1 ± 0.0	0.373
Te_N_, s	2.2 ± 0.2	2.4 ± 0.2	2.7 ± 0.3	2.4 ± 0.2	0.129
TiN/TtN, %	37.0 ± 1.4	33.4 ± 1.6	35.7 ± 1.7	36.5 ± 1.5	0.042
Peak EAdi, μV	12.9 ± 1.7	12.0 ± 1.5	12.8 ± 1.9	12.4 ± 1.9	0.611
Peak EAdi, μV	8.1 ± 5.5	9.3 ± 6.1	8.0 ± 2.4	6.8 ± 2.0	0.864

*Data are provided as mean ± SD*.

*PS_10_, pressure support ventilation with cycling-off criteria set to 10%; PS_30_, pressure support ventilation with cycling-off criteria set to 30%; PS_50_, pressure support ventilation with cycling-off criteria set to 50%; PS_AUTO_, pressure support ventilation with automatic adjustment system; Ppeak, peak airway pressure; PEEP, positive end expiratory pressure; Vt, tidal volume; RR, respiratory rate; Ti_N_, neural inspiratory time; Te_N_, neural expiratory time; Peak EAdi, peak diaphragm electrical activity*.

*Compared with PS_10_*,

a *P < 0.05; Compared with PS_30_*.

## Discussion

This study showed that when compared with PSV with prefixed pneumatic controllers, an automatic adjustment system decreased total AI and improved patient-ventilator interaction mainly through a decrease of micro-asynchronies. The automatic system was associated with the lower cycling-off error, triggering error, and triggering effort in PSV patients.

### AI and NeuroSync Index

Both AI and the NeuroSync index are indicators that reflect the overall patient-ventilator interaction from different perspectives. PS_AUTO_ constantly reduced total AI and the NeuroSync index when compared with PSV with prefixed pneumatic controllers, indicating improved patient-ventilator interaction. Given that macro-asynchronies were rare, the benefit of PS_AUTO_ in reducing the total AI was mainly due to the reduction of micro-asynchronies. These findings agree with previous work comparing PSV and neurally adjusted ventilatory assist, which showed the difference in AI is found only in micro-asynchronies (14).

The present study showed a higher total AI (median of 23–57% during PS_10_, PS_30_, PS_50_, and PS_AUTO_) when compared with those in previous studies (range from 0 to 27%) (6, 16, 17). Despite the differences among study patients and ventilators used, the major reason for the apparent differences between studies might relate to the calculation method for the AI. First, inspiratory trigger delay was included in the calculation of the AI in the present study, which provided about one-third to one-half of the total AI during PSV with prefixed pneumatic controllers. However, the previous study did not calculate inspiratory trigger delay in the AI (6). Second, we defined asynchrony as an error of 200 ms between the origin of the EAdi and ventilator insufflation, which was more sensitive than the threshold used in previous studies (6, 16–18). Therefore, the AI in the present study is more sensitive and comprehensive and therefore not comparable to those in other studies.

### Cycling-Off Error

Cycling-off asynchrony is dependent on factors, such as the inspiratory effort, neural inspiratory time, assist levels, the time constant of the respiratory system, and cycling-off criteria of the patients (3). Consequently, the optimum flow cycling-off criteria vary among patients and can range from very low levels (5%) in patients with a restrictive condition (such as acute respiratory distress syndrome) (4, 19) to more than 50% in patients with an obstructive condition (such as chronic obstructive pulmonary disease) (5, 20, 21). A previous study showed in a mixed sample of patients that the use of a variable, real-time-adjusted termination criterion improved some indices of patient-ventilator asynchrony when compared with a fixed termination criterion (5% of peak inspiratory flow) (22). However, a termination criterion of 5% of peak inspiratory flow was not commonly used clinically during PSV. Our results showed a significant improvement in relative cycling-off error during PS_AUTO_ when compared with PSV with prefixed cycling-off criteria of 10%, 30%, and 50%. It was not unexpected that during PSV with prefixed pneumatic controllers, PS_50_ and PS_10_ were the “best” cycling-off settings with the lowest relative cycling-off errors in the obstructive and restrictive subgroups. In each subgroup, relative cycling-off error in PS_AUTO_ was comparable with the “best” cycling-off setting during PSV with a prefixed cycling-off.

### Triggering Error

The present study showed the median delay for triggering during PS_10_, PS_30_,PS_50_, and PS_AUTO_ ranged from 187 to 130 ms. These values fall within the 80–540 ms range of values previously reported for PSV (1, 18, 23). Beloncle et al. reported absolute values for trigger delay <200 ms in almost all patients, which was lower than that reported in the present study (18). The different ventilators and flow-trigger used in different studies might be one reason, and different types of the enrolled patients might be another reason for the difference in trigger delay. During PS_AUTO_, the algorithm will trigger the ventilator to initiate the inspiratory phase when it detects a sudden increase of flow waveform, which reflects the inspiratory effort, leading to a reduced triggering delay. Furthermore, triggering delay was likely reduced as a consequence of reduced cycling-off delay during PS_AUTO_, which led to a longer expiration time and lower PEEPi, especially in patients with obstructive conditions (5). Unfortunately, we did not measure PEEPi during each mode.

Of note, a single flow-trigger level in the present study made it hard to draw conclusions regarding the effect of the PS_AUTO_ mode on inspiratory triggering asynchronies when compared with lower flow-triggering (e.g., 1.0 L/min). From this perspective, a fixed flow-trigger of 1.5 L/min might be not sensitive enough. Considering the similar or shorter triggering delay and no obvious auto-triggering during PS_AUTO_, automatic adjustment of triggering based on waveforms might be a useful tool for making the triggering setting easier.

### Inspiratory Effort and Breathing Pattern

Because PS_AUTO_ significantly reduced triggering delay, it was not unexpected that it was associated with lower inspiratory effort for triggering. The present study showed a comparable breathing pattern and respiratory drive between PS_AUTO_ and PSV with prefixed pneumatic controllers. Of interest, neural expiratory time remained unchanged at the various cycling-off settings in the present study. These findings agree with previous work in which expiratory time did not change with the increase in cycling-off criteria in chronic obstructive pulmonary disease patients (5, 20). However, the findings contradict those in previous studies which show an increased expiratory time in the presence of delayed cycling in acute lung injury (24, 25). Therefore, PS_AUTO_ improved the cycling-off criteria, which was demonstrated to affect the inspiratory time only at high-pressure support (20). Peak EAdi around 12 μV confirmed the absence of over-assistance during PSV in the present study.

### Limitations

There are some limitations that should be noted. First, our study was conducted in a small group of patients. Second, respiratory mechanics were evaluated in patients under sedation who were not actively breathing, therefore, the results will be different from those measured during PSV. Third, patients were maintained at each mode setting for only 20 min, and steady-state conditions might not have been achieved. However, the duration was in line with that of several studies on the effects of cycling criteria modifications (4, 19).

## Conclusions

An automatic adjustment system based on waveform was associated with less patient-ventilator asynchrony when compared with PSV with prefixed pneumatic controllers. Our results indicated that this system might be a useful tool to titrate more personalized mechanical ventilation, especially in patients with a high risk of patient-ventilator asynchrony.

## Data Availability Statement

The raw data supporting the conclusions of this article will be made available by the authors, without undue reservation.

## Ethics Statement

The studies involving human participants were reviewed and approved by Institutional Ethics Committee of Zhongda Hospital. The patients/participants provided their written informed consent to participate in this study.

## Author Contributions

LL, YYa, DC, and HQ have given substantial contributions to the conception or the design of the manuscript. LL, YYu, XX, and QS to acquisition, analysis, and interpretation of the data. All authors contributed equally to the manuscript and read and approved the final version of the manuscript.

## Funding

This study was supported by the Clinical Science and Technology Specific Projects of Jiangsu Province [BE2020786 and BE2019749], the National Natural Science Foundation of China [Grant Numbers 81870066 and 81670074], the Natural Science Foundation of Jiangsu Province (BK20171271), Jiangsu Provincial Medical Youth Talent (QNRC 2016807), and Third Level Talents of the 333 High Level Talents Training Project in the fifth phase in Jiangsu (LGY2016051). This study received funding from Mindray (China). The funder was not involved in the study design, collection, analysis, interpretation of data, the writing of this article, or the decision to submit it for publication.

## Conflict of Interest

LL and HQ received a grant from Mindray (China). The remaining authors declare that the research was conducted in the absence of any commercial or financial relationships that could be construed as a potential conflict of interest.

## Publisher's Note

All claims expressed in this article are solely those of the authors and do not necessarily represent those of their affiliated organizations, or those of the publisher, the editors and the reviewers. Any product that may be evaluated in this article, or claim that may be made by its manufacturer, is not guaranteed or endorsed by the publisher.
