# Supplementary material for: Automatic Adjustment of the Inspiratory Trigger and Cycling-Off Criteria Improved Patient-Ventilator Asynchrony During Pressure Support Ventilation
Source: Front Med (Lausanne). 2021 Nov 12;8:752508. doi: 10.3389/fmed.2021.752508 (PMC8632800; doi:10.3389/fmed.2021.752508)
Supplement: Supplementary file 1 [file Data_Sheet_1.PDF]

Supplementary Material

1 Supplementary Data

1.1 Working principle of the automatic adjustment system (IntelliCycle^{TM}2.0)

The automatic adjustment system technology aims to automatically identify the start of patient spontaneous inspiration and expiration.

1.1.1 Waveform guided detection of patient inspiration: The automatic adjustment system analysis flow and pressure waveform thousand times per second. By the real-time analyzing of waveform shapes and slope, the patient inspiratory effort doesn't need to inhale the flow to positive value (for example 2L/min). The algorithm will trigger the ventilator to inspiratory phase when it detects a suddenly increase of flow waveform, which reflect the inspiratory effort. This technology can decrease the delay of inspiratory trigger and non-effective trigger. Especially for chronic obstructive pulmonary disease (COPD) patients, sometime their inspiratory effort is not strong enough to inhale the flow to positive value. In this case the conventional flow or pressure trigger may cause many non-effective triggering (Figure S1).

1.1.2 Auto adjustment of expiration cycling-off threshold: Usually the expiration cycling-off threshold is 25% or set by doctors according to the patient need. The automatic adjustment system can automatically detect the delay or ahead of expiration cycling of individual patient, then can adjust the cycling-off criteria automatically according to the degree of delay or ahead. When late cycling-off occurred, the airway pressure will overshoot at the end of inspiration, or the flow waveform will suddenly decrease. By real-time analysis of pressure-time and flow-time waveform, the algorithm can automatically detect the late cycling-off When premature cycling-off occurred, the flow waveform may present non-monotone pattern because of patient inspiratory effort (Figure S2). When late cycling-off is detected, the algorithm will increase the cycling-off criteria and when premature Cycling-off is detected, the algorithm decreases the cycling-off criteria. Finally, the algorithm can find the optimum cycling-off criteria for the individual patient, without doctors involved.

1.2 Respiratory mechanics measurement

Patients were initially on volume control ventilation (VCV) at tidal volume (VT) 6 mL/kg predicted body weight (PBW), inspiratory flow of 30 L/min, and mandatory breathing frequency and positive end-expiratory pressure (PEEPe) matching that observed during PSV before sedation. To suppress the spontaneous drive to breath (abolish electrical activity of the diaphragm (EAdi), patients received continuous intravenous (IV) sedation by propofol up to the dose of 2 mg/kg/h. If at this propofol dose the respiratory drive was not totally suppressed, remifentanil was also infused at

the dose of 6-15 μg/kg/h just before the measurement of compliance, resistance and intrinsic positive end expiratory pressure (PEEPi).

Three seconds of Inspiratory and expiratory hold were performed to measure plateau pressure and total PEEP, respectively. Static compliance of respiratory system (C_{RS}) was calculated from the formula: Tidal volume / (Plateau pressure-total PEEP). R_{RS} Resistance of respiratory system (R_{RS}), was calculated from the formula: Resistance = (Peak pressure -Plateau pressure) / Flow. PEEPi was assessed during VCV at PEEPe of zero using the end-expiratory airway occlusion method.

1.3 Data obtain and analysis

Trigger and cycling-off error: Cycling-off error was calculated as the time difference between time points for early decrease in airway pressure and 70 % of peak EAdi. Trigger error was measured as the time difference between the onset of the EAdi and the early initial rise in Paw. Relative trigger and cycling-off error were calculated as the absolute value of percentages of neural inspiratory and expiratory time periods, respectively. Events where EAdi and Paw were completely dissociated, such as wasted efforts, auto-triggering, double-triggering were assigned 100% error. Relative trigger and cycling-off error were calculated as the percentages of neural inspiratory and expiratory time periods, respectively.

1.4 Breathing pattern

Neural inspiratory time (Ti_N) was calculated between the onset of EAdi and the return to 70 % of peak EAdi.[19] Neural expiratory time (Te_N) was calculated as the time between the return to 70 % of peak EAdi and the onset of the next EAdi.

1.5 Inspiratory effort and inspiratory effort for triggering

Pes-time product (PTP_{es}) was used to estimate the inspiratory effort, which was measured by the area under the Pes signal between the onset of EAdi and the end of inspiratory flow in one minute. Pre-trigger Pes-time product ($PTP_{es\text{-}trig}$) was used to estimate the inspiratory effort for triggering, which was measured by the area under the Pes signal between the onset of EAdi and the start of inspiratory flow in one minute.

2 Supplementary Figures and Tables

2.1 Supplementary Figures

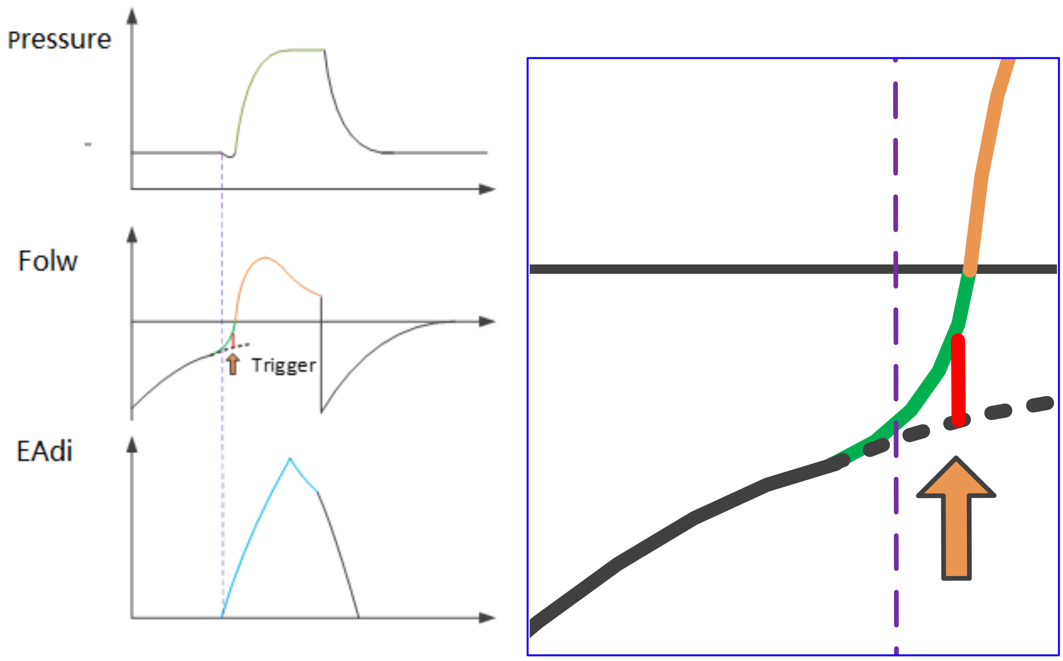

Supplementary Figure 1. automatic adjustment during trigger

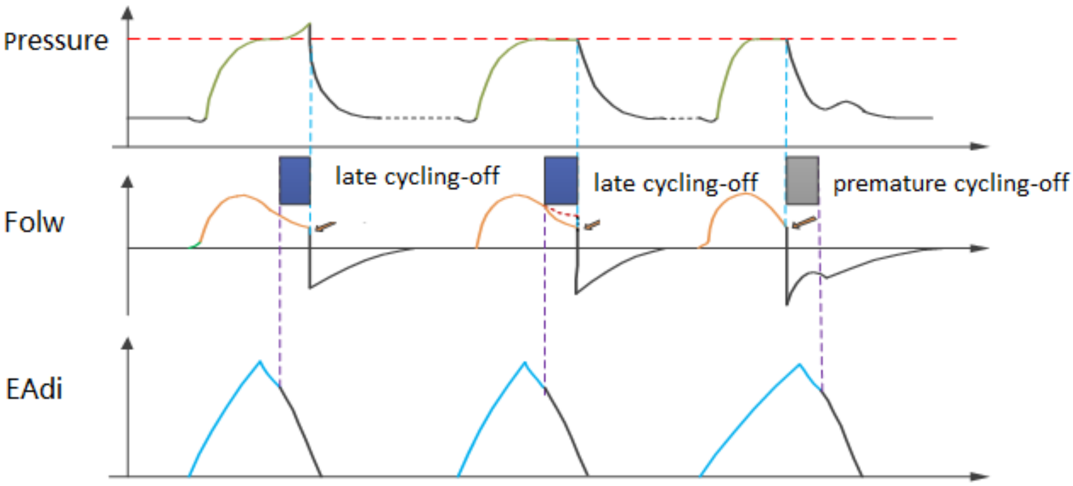

Supplementary Figure 2. automatic adjustment during cycling-off

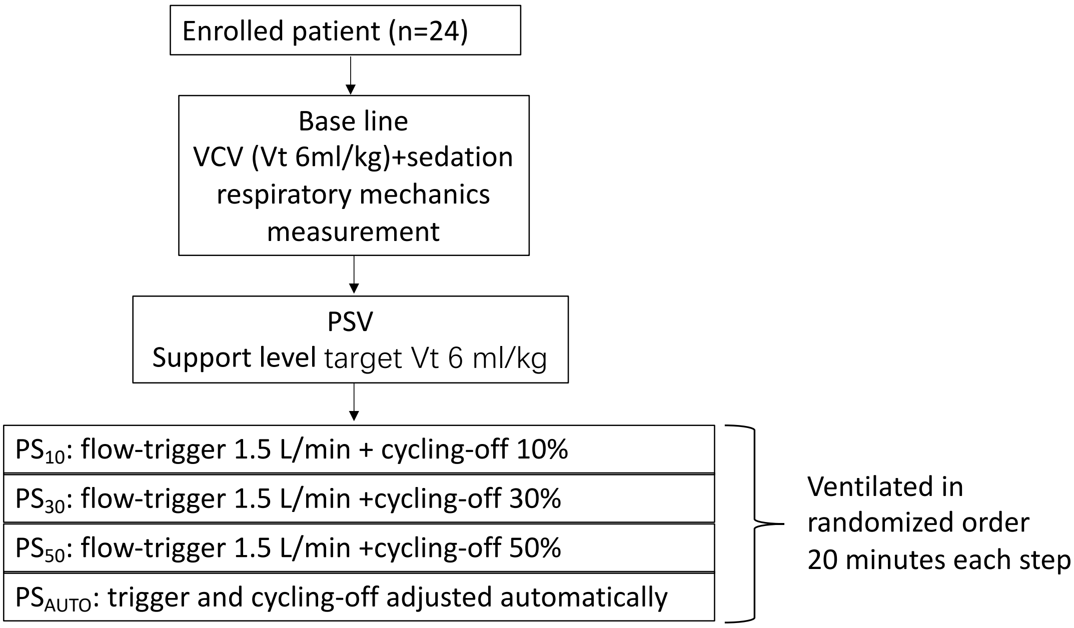

Supplementary Figure 3. Flow chart of the study protocol.

PS_{10}, pressure support ventilation with cycling-off criteria set to 10%; PS_{30}, pressure support ventilation with cycling-off criteria set to 30%; PS_{50}, pressure support ventilation with cycling-off criteria set to 50%; PS_{AUTO}, pressure support ventilation with automatic adjustment system; VCV, volume control ventilation; Vt, tidal volume.

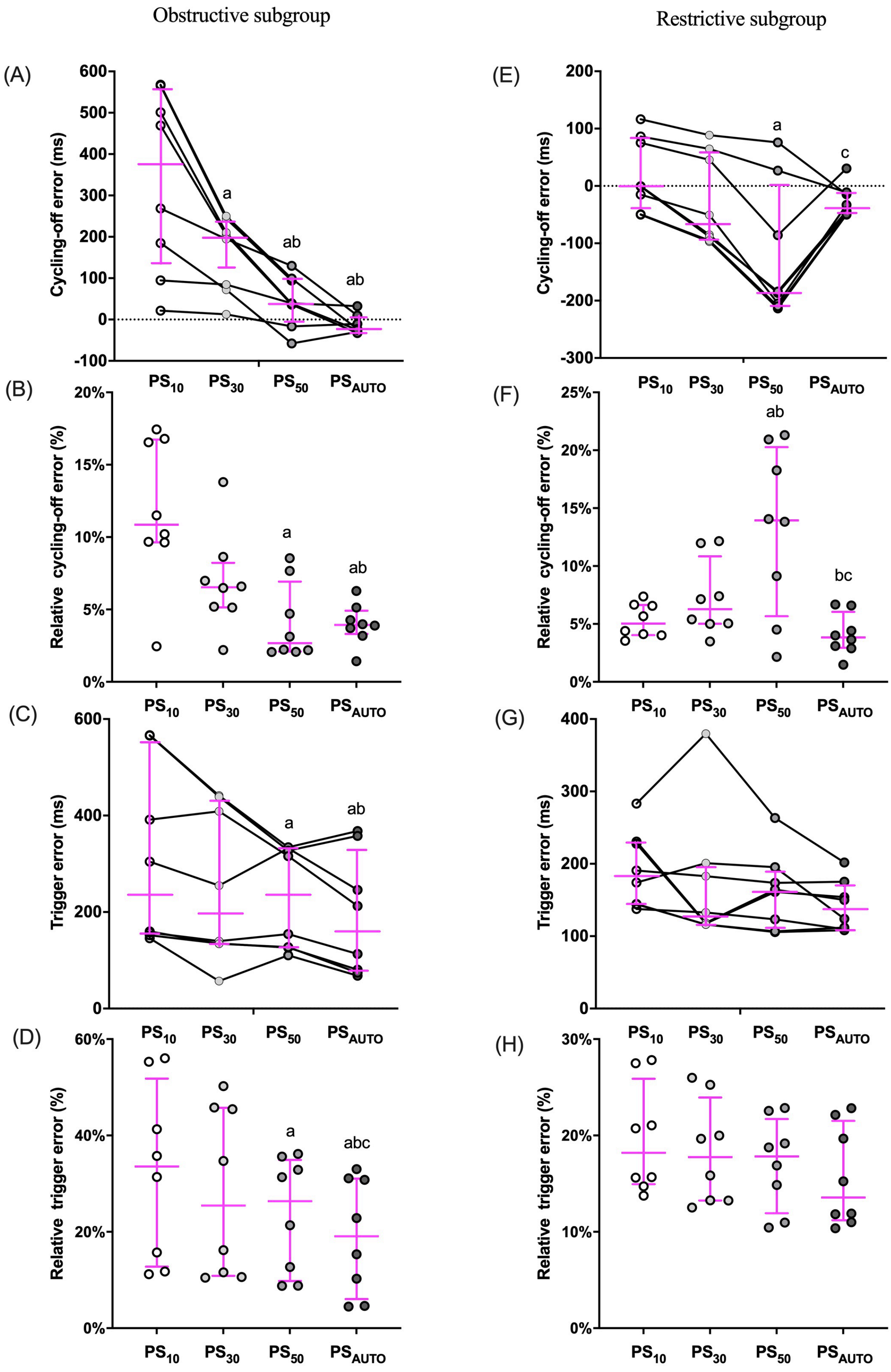

Supplementary Figure 4. cycling-off error and trigger error in subgroups.

A cycling-off error in obstructive subgroup, **B** relative cycling-off error in obstructive subgroup, **C** trigger error in obstructive subgroup, D relative trigger error in obstructive subgroup, **E** cycling-off error in restrictive subgroup, **F** relative cycling-off error in restrictive subgroup, **G** trigger error in restrictive subgroup, **H** relative trigger error in in restrictive subgroup.

Y-axis for cycling-off error: positive values indicate late cycling off, and negative values indicate early cycling off. Magenta line showed median (interquartile range).

ms, millisecond; PS_{10}, pressure support ventilation with cycling-off criteria set to 10%; PS_{30}, pressure support ventilation with cycling-off criteria set to 30%; PS_{50}, pressure support ventilation with cycling-off criteria set to 50%; PS_{AUTO}, pressure support ventilation with automatic

Compared with PS_{10}, $^{a}P < 0.05$; Compared with PS_{30}, $^{b}P < 0.05$; Compared with PS_{50}, $^{c}P < 0.05$

2.2 Supplementary Tables

Supplementary Table 1. Randomization order of ventilation modes in each patient

Patient No.	Modes			
1	PS_{30}	PS_{10}	PS_{50}	PS_{AUTO}
2	PS_{50}	PS_{30}	PS_{10}	PS_{AUTO}
3	PS_{10}	PS_{50}	PS_{AUTO}	PS_{30}
4	PS_{10}	PS_{50}	PS_{30}	PS_{AUTO}
5	PS_{10}	PS_{AUTO}	PS_{50}	PS_{30}
6	PS_{AUTO}	PS_{50}	PS_{30}	PS_{10}
7	PS_{10}	PS_{30}	PS_{50}	PS_{AUTO}
8	PS_{AUTO}	PS_{10}	PS_{30}	PS_{50}
9	PS_{30}	PS_{50}	PS_{AUTO}	PS_{10}
10	PS_{10}	PS_{30}	PS_{50}	PS_{AUTO}

11	PS_{10}	PS_{50}	PS_{30}	PS_{AUTO}
12	PS_{AUTO}	PS_{50}	PS_{10}	PS_{30}
13	PS_{50}	PS_{30}	PS_{10}	PS_{AUTO}
14	PS_{10}	PS_{30}	PS_{50}	PS_{AUTO}
15	PS_{10}	PS_{AUTO}	PS_{30}	PS_{50}
16	PS_{AUTO}	PS_{10}	PS_{30}	PS_{50}
17	PS_{30}	PS_{AUTO}	PS_{50}	PS_{10}
18	PS_{AUTO}	PS_{50}	PS_{10}	PS_{30}
19	PS_{AUTO}	PS_{30}	PS_{10}	PS_{50}
20	PS_{AUTO}	PS_{10}	PS_{50}	PS_{30}
21	PS_{10}	PS_{AUTO}	PS_{30}	PS_{50}
22	PS_{10}	PS_{50}	PS_{AUTO}	PS_{30}
23	PS_{10}	PS_{50}	PS_{30}	PS_{AUTO}
24	PS_{10}	PS_{30}	PS_{50}	PS_{AUTO}

$PS_{10,}$ pressure support ventilation with cycling-off criteria set to 10%; $PS_{30,}$ pressure support ventilation with cycling-off criteria set to 30%; $PS_{50,}$ pressure support ventilation with cycling-off criteria set to 50%; $PS_{AUTO,}$ pressure support ventilation with automatic.

Supplementary Table 2. Asynchronies, NeuroSync index and inspiratory effort in subgroup

Patient	Parameters	PS_{10}	PS_{30}	PS_{50}	PS_{AUTO}	P value
Obstructive subgroup (n=8)	Total AI, %	107.0(77.6, 127.7)	95.0(46.6, 104.3)	56.0(16.5, 68.0) [a]	39.0(5.1, 45.5) [ab]	<0.001
	Macro AI	6.0 (0.0, 23.4)	10.0 (0.0, 12.0)	1.0 (0.0, 7.7)	0.0 (0.0, 2.9)	0.308
	Micro AI	107.0(67.7, 121.1)	91.0(39.1, 100.3)	56.0 (165.3, 65.0)	39.0(4.4, 44.3) [ab]	<0.001
	Ineffective triggering, %	3.7 (0.0, 7.7)	0.0 (0.0, 7.3)	0.0(0.0, 1.0)	0.0(0.0, 0.0) [a]	0.038
	Auto-triggering, %	0.0(0.0, 2.2)	0.0(0.0, 0.9)	0.0(0.0, 0.0)	0.0(0.0, 0.0)	0.261
	Double triggering, %	0.0(0.0, 11.0)	0.0(0.0, 12.0)	0.0(0.0, 5.6)	0.0(0.0, 1.8)	0.112
	Premature cycling-off, %	0.0(0.0, 0.0)	0.0(0.0, 0.0)	0.0(0.0, 1.1)	0.0(0.0, 1.8)	0.453
	Late cycling-off, %	41.3(11.0, 82.0)	32.8(0.9, 58.4)	0.0(0.0, 0.9) [a]	0.0(0.0, 2.2) [a]	<0.001
	Inspiratory trigger delay,%	38.7(20.2, 72.2)	35.5(19.9, 70.3)	35.8(15.4, 67.3)	19.1(1.2, 52.0) [ab]	0.004
	NeuroSync index, %	22.5±11.3	17.5±10.0[a]	13.8±6.6[ab]	11.5±6.2[abc]	0.001

	$PTP_{es\text{-}Trig}$, $cmH_2O.S.min^{-1}$	-3.3(-11.7, -1.0)	-2.8(-13.7, -1.2)	-2.4(-9.6, -1.1)	-1.8(-7.4, -0.9) ^{abc}	0.001
	PTP_{es}, $cmH_2O.S.min^{-1}$	-20.4(-90.0, -13.8)	-29.5(-99.5, -10.6)	-33.3(-51.8, -8.2)	-26.3(-68.7, 7.8)	0.682
Restrictive subgroup (n=8)	Total AI, %	41.0(14.8, 69.0)	29.0(23.0, 41.8)	53.0(49.6, 53.5)	38.0(31.9, 38.9) ^{c}	0.015
	Macro AI	0.0(0.0, 4.9)	0.0(0.0, 6.3)	0.0(0.0, 3.3)	0.0(0.0, 5.0)	0.972
	Micro AI	41.0 (13.4, 54.8)	25.0(17.2, 38.4)	51.0 (48.3, 52.1)	26.1 (1.8, 30.1) ^{c}	0.008
	Ineffective triggering, %	0.0(0.0, 2.2)	0.0(0.0, 0.0)	0.0(0.0, 1.2)	0.0(0.0, 2.3)	0.250
	Auto-triggering, %	0.0(0.0, 0.0)	0.0(0.0, 11.0)	0.0(0.0, 1.2)	0.0(0.0, 0.0)	0.145
	Double triggering, %	0.0(0.0, 8.9)	0.0(0.0, 0.0)	0.0(0.0, 0.0)	0.0(0.0, 8.9)	0.392
	Premature cycling -off, %	1.2(0.0, 2.6)	0.9(0.0, 9.1)	35.0(3.3, 49.3)	0.0(0.0, 0.0) ^{c}	0.011
	Late cycling-off, %	0.0(0.0-6.2)	0.7(0.0-5.4)	0.0 (0.0-0.0)	0.0(0.0-0.0)	0.055
	Inspiratory trigger delay, %	38.9(6.1, 45.2)	19.4(0.4, 35.5)	16.7(0.9, 44.)	24.8(1.4, 34.4)	0.069
	NeuroSync index, %	12.5±2.7	12.7±2.4	15.0±2.6^{ab}	9.92±2.7 ^{abc}	<0.001

	$PTP_{es-Trig}$, $cmH_2O.S.min^{-1}$	-1.7(-5.6, -0.8)	-1.4(-2.7, -0.7) [a]	-1.4(-2.3, -0.5) [a]	-1.3(-2.6, -0.6) [a]	0.001
	PTP_{es}, $cmH_2O.S.min^{-1}$	-38.9(-81.0, -1.1)	-24.5(-81.8, -0.8)	-25.0(-71.9, -0.8)	-24.4(80.2, -1.5)	0.789

Data are provided as mean ± SD or median (interquartile range).

NeuroSync index is an overall indicator of patient–ventilator interaction, where 0% error = perfect and 100% error = zero patient–ventilator interaction; $PTP_{es-Trig,}$ Pre-trigger Pes-time product; $PTP_{es,}$ Pes-time product; $PS_{10,}$ pressure support ventilation with cycling-off criteria set to 10%; $PS_{30,}$ pressure support ventilation with cycling-off criteria set to 30%; $PS_{50,}$ pressure support ventilation with cycling-off criteria set to 50%; $PS_{AUTO,}$ pressure support ventilation with automatic adjustment system.

Compared with PS_{10}, $^{a}P < 0.05$; Compared with PS_{30}, $^{b}P < 0.05$; Compared with PS_{50}, $^{c}P < 0.05$

Supplementary Table 3. Breathing pattern and respiratory drive in obstructive and restrictive subgroups

Patient	Parameter	PS_{10}	PS_{30}	PS_{50}	PS_{AUTO}	P value
obstructive subgroups (n=8)	Ppeak, cmH_2O	20.4±3.5	19.6±3.4	19.2±3.5 [a]	17.2±5.3[a]	0.001
	PEEP, cmH_2O	6.0±2.4	5.9±2.3	6.0±2.3	6.9±2.0	0.051
	Vt, cmH_2O/kg	6.1±0.2	6.0±0.2	6.0±0.1	6.0±0.1	0.482
	RR_N, breath/min	19.4±8.2	21.1±10.6	19.3±7.9	20.4±9.1	0.290
	Ti_N, s	1.1±0.2	1.0±0.2 [a]	1.1±0.1	1.0±0.2	0.051
	Te_N, s	2.6±1.3	2.8±1.5	3.0±1.7	2.9±1.4	0.136
	TiN/TtN, %	34.0±9.2	32.3±8.4	32.7±9.2	32.3±8.5	0.930
	Peak EAdi, μV	6.0±2.4	5.8±2.3	6.0±2.3	6.9±2.1	0.241
restrictive subgroup (n=8)	Ppeak, cmH_2O	15.6±3.1	14.7±3.1	15.6±2.9	14.8±3.1	0.136
	PEEP, cmH_2O	6.9±1.5	6.8±1.4	6.8±1.4	6.8±1.4	0.272
	Vt, cmH2O/kg	6.1±0.2	6.2±0.3	6.1±0.3	6.0±0.1	0.492
	RR_N, breath/min	22.4±3.0	20.2±6.7	19.9±5.1	22.2±5.0	0.583
	Ti_N, s	1.1±0.2	1.1±0.2	1.1±0.2	1.1±0.2	0.136
	Te_N, s	1.7±0.3	2.4±1.4	2.3±1.2	1.8±0.5	0.175
	TiN/TtN, %	40.7±4.3	35.0±8.5	36.1±7.1	39.5±3.1	0.105
	Peak EAdi, μV	16.7±8.7	15.2±8.0	17.6±11.9	18.1±10.1	0.369

Data are provided as mean ± SD.

PS_{10}, pressure support ventilation with cycling-off criteria set to 10%; PS_{30}, pressure support ventilation with cycling-off criteria set to 30%; PS_{50}, pressure support ventilation with cycling-off criteria set to 50%; PS_{AUTO}, pressure support ventilation with automatic adjustment system; Ppeak, peak airway pressure; PEEP, positive end expiratory pressure; Vt, tidal volume; RR, respiratory rate; Ti_N, neural inspiratory time; Te_N, neural expiratory time; Peak EAdi, peak diaphragm electrical activity.

Compared with PS_{10}, $^{a}P < 0.05$; Compared with PS_{30}, $^{b}P < 0.05$; Compared with PS_{50}, $^{c}P < 0.05$
